# 

**Published:** 1887-12-17

**Authors:** 


### NOTICE TO ADVERTISERS.

The scale of charges for small prepaid Advertisements—Situations Wanted, Situations Vacant, &c., &c., is as follows:—

| | s. | d. |
|---|---|---|
| One insertion of three lines | 1 | 0 |
| Three " " " | 2 | 6 |
| Per line afterwards | 0 | 6 |

*A line averages ten words.*

All copy for Advertisements must be sent to A. P. Watt, 2, Paternoster Square, E.C., by first post on the Tuesday *preceding* publication.

### NOTICE TO CORRESPONDENTS.

All correspondence must be written on one side of the paper only.

We cannot undertake to return MSS. not used.

**Communications,** whether intended for insertion or for private information, must be authenticated by the names and addresses of the writers, not necessarily for publication.

**Local papers** containing reports or news paragraphs **should be marked.**

It is especially requested that early intelligence of **local events of interest,** or which it may be thought desirable to bring under notice, may be sent direct to the Editor.

**All communications respecting editorial matters** should be addressed to the Editor, Norfolk House, Norfolk Street, Strand, London, W.C.

### THE STUDENTS' COLUMN.

### SOCIETY OF APOTHECARIES OF LONDON.

The following questions were given at the recent Examinations of the Ap thecaries Society:—

### EXAMINATION IN SURGERY.

Surgery.—Four questions to be answered. 1. Give the signs of fracture of the shaft of the Femur, and describe the treatment you would adopt; 2. Give the diagnostic signs, treatment, and prognosis of a case of Hæmatocele; 3. Give the symptoms, diagnosis, prognosis, and treatment of Cutaneous Erysipelas; 4 Give the symptoms of Strangulated Femoral Hernia, and describe the treatment you would adopt, including an account of the operation of Herniotomy; 5. Give the causes, symptoms, treatment, and prognosis of purulent Ophthalmia.

Surgical Pathology.—One question to be answered. 1. Describe the mode of formation, and mode of cure of a Sacculated Aneurism; 2. Describe the changes that take place in the Synovial Membrane in Tubercular Disease of a joint.

Surgical Anatomy.—One question to be answered. 1. Describe the Palmar Arches. 2. What structures would be cut through in the operation of Excision of the Eyeball.

### EXAMINATIONS IN MEDICINE.

Medicine.—Four questions to be answered. 1. Give an account of Asthma, to include its pathology, symptoms, physical signs, diagnosis, and treatment; 2. The area of Cardiac dulness being found increased, describe how you would proceed to decide as to whether hypertrophy or dilatation of the Heart, or pericardial effusion were the cause; 3. What are the cautses of Polyuria? Give the diagnosis between the several va rieties, and he treatment for any one form; 4. Give the etiology, diagnosis, and treatment of Laryngismus Stridulus; 5. Give the symptoms, course, duration, mode of death, and pathology of Glosso-labio-laryngeal Paralysis; 6. Describe a case of confluent Small-pox from its commencement to a favourable termination, giving the dates of the various stages, and the treatment you would follow.

Pathology.—Two questions to be answered. 1. Describe the different forms of Sarcoma, and state how they differ from Carcinoma; 2. Give an account of the post-mortem appearances which may be found as a result of Tertiary Syphilis; 3. Describe the various forms of Hypertrophy and dilatation of the Heart respectively, and state how they are caused.

Therapeutics.—One question to be answered. 1. Give the dietary you would prescribe (*a*) in Diabetes Mellitus; (*b*) in Intra-thoracic Aneurism; (*c*) in Chronic Gout; (*d*) in a Rickety Child two years of age; 2. How would you treat Insomnia (*a*) in Delirum Tremens; (*b*) in Typhus Fever; (*c*) in Heart Disease; (*d*) in Bright's Disease?

Forensic Medicine and Toxicology.—Three questions to be answered. 1. Give the symptons of poisoning by Prussic Acid, Cyanide of Potassium, and Bicyanide of Mercury, respectively. How would you detect Prussic Acid in the contents of the stomach? 2. What are the chemical and physiolog cal antidotes for Morphine, Strychnine, and Atropine respectively? 3. What evil effects result from over crowding in hospitals, and what amount of of cubic space of air do you consider necessary for a sick person? 4. How is death caused by drowning? What P M. appearances would you expect to find? What plans of treatment have been proposed for the restoration of the apparently drowned?

The following gentlemen having passed the Qualifying examination in Medicine, Surgery, and Midwifery, have received Certificates entitling them to practise in the same, and have been admitted as Licentiates of the Society: Beckett, Louis, London Hospital; Bulger, Alfred James Queen's College, Birmingham; Bokenham, Thomas Jessopp, St. Bartholomew's; Cunningham, John, Liverpool School of Medicine; Flux, George Belben, King's College; Fullard, John, Queen's College, Birmingham; Snape Ernest Alfred, Charing Cross; Spong, Harry, Leeds School of Medicine.

The following gentlemen passed the Examination in Surgery: Coulton, John James, London Hospital; Cox, William Alfred Spencer, Owens College, Manchester; Cunningham, John, Liverpool Royal Infirmary; Evans, Evan, St. Bartholomew's; Hayward, Gerald Cobden, Middlesex; Hicks, John Sydney, London Hospital.

### Amusements with Profit for all Ages.

**Rules.**—All answers must be addressed to the Prize Editor, The Hospital, Norfolk House, Norfolk Street, Strand, W.C., not later than the *first* post on Thursday next. The full name and address must in all cases be given, but a *nom de plume* may be added for publication. Only one side of the paper must be written on.

### FOR MEN AND WOMEN.

### Work Competition.

I. A PRIZE of THREE GUINEAS will be given to the competitor who sends in the best specimen of painting, which may take the form of a picture, panel, screen, flower-pots, etc., or illuminations.

II. TWO GUINEAS will be given for the best dressed doll or other piece of work suitable for presentation to the inmates of the hospitals.

III. ONE GUINEA will be given for the best scrap-book.

All contributions to the above must sent in by December 31st, addressed "Work Competition," care of Editor, The Lodge, Porchester Square, London, W.

We have received a doll from Nurse Hall, and several other packages.

### Acrostic Competition.

A PRIZE of ONE GUINEA will be given to the competitor who gains the highest number of marks for best original acrostics during the ensuing quarter. All acrostics sent in for this competition will be credited according to merit, twelve marks being the highest score given for one acrostic.

A PRIZE of HALF-A-GUINEA to be given to the competitor who gains the highest score for solution of acrostics set, including the one inserted on November 26th. One mark only will be given to the competitors who solve all the lights of each acrostic.

These competitions to alternate weekly.

This week credit will be given for the best *Original Double Acrostic in verse* (No 2.)

Results of *First* Original Double Acrostic. The acrostics sent in are, on the whole, above the average, but, as will be seen by the appended list of names, no one has succeeded in gaining the highest score (12 marks). We hope therefore that this week competitors will do their best to make the second and other acrostics—which are to follow fortnightly—eclipse their first essays.

Answers have been received from Mater (11), Brownie (11), E. M. B. (11), Pasteur (11), Ellice (10), Padre (10), H. M. (9), Gretta (9), Condorcet (9), E. E. (8), Toby (8).

### II.—Word and Literary Competition.

Commenced November 5th, 1887; ends January 28th, 1888.

Three prizes of one guinea, 15*s*. and 10*s*. 6*d*. will be given each quarter to the three competitors who obtain the highest number of marks: (1) by making the largest number of words derived from the word or phrase set for anatomical dissection; or (2) for the best answers to (6); or (3) by (1) and (2) combined.

(A) THE WORD COMPETITION.

We shall give the newspapers for dissection during this quarter, that for this week being "The Globe."

Observe.—Proper names, abbreviations, foreign words, words of less than four letters and repetitions are barred. Nuttall's *Standard* dictionary only to be used.

### Results of Fifth Week.

Reldas (145), E. E. (137), Ellice (134), Gretta (131), Lauriston (130), Skye (128), Tabs (127), Brisbane (125), Tim (124), Kath (107), Toby (105), K. M. (104), Condorcet (97), Brownie (60).

(B) THE LITERARY COMPETITION.

For particulars of this see page 157, Nov. 26th. *Gretta* sends a letter from India.

### THE CHILDREN'S CORNER.

For Prizes see page iv. of last week's issue.

### Puzzles—Third Week.

No. 9. Double Acrostic. 1. A winter amusement in Scotland. 2. Something always welcome, especially at this time of year. 3. An unwelcome command. 4. An agreeable summons to young and old. 5 Set the Christmas pudding alight with this. 6. One of the Society Islands. 7. When *I* appear the dance begins. 8. Protection from sun or rain. 9. What I wish you all. Initials and finals form seasonable salutations.

No. 10. Numerical Charade. My 14, 21, 3, 17, 5, what all desire. My 18, 12, 7, 1, 9, 10, a river. My 11, 15, 8, 4, a verb. My 6, 2, 19, 3, "Fair Luna." My 11, 7, 16, 13, 10, found amongst corn. My 20 and 2 depart.

No. 11. Enigma.

By fingers fair my *first* is formed.
A little pronoun is my *second*.
And half of it my *whole* is reckoned.

No. 12. Geographical Charade. My *first* is an island in the Irish Sea. My *second* and *third* the name of a very old English city. My *whole* is a town in Lancashire.

### Results of First Week Puzzles.

No. 1. Towns in America Enigmatically Expressed.—1. Louis-ville=Louisville. 2. Hamill ton=Hamilton. 3. Ball Tim-ore=Baltimore. 4. Brook-lynn=Brooklynn.

No. 2. Riddle-me-ree.—Ceylon.

No. 3. Puzzle Sentence.—A very fine day.

No. 4.—Arm Ada=Armada.

*All right*—Scrooge, A. C. K., Pepita, Military Cadet, Holmes, Skye, Ossian, M. S. W., Squeaker, Excelsior, H. E. W., Jumps, Alma, A. G. S., McCulloch, Mount Edgecumbe, Plummy, Fluff, Alsie, Gem, Toby, Tim. *Three right*—Spider, Poodle, Happy Eliza, Judy, Snap. *Two right*—Fern.

### Notices to Correspondents.

*C. M. N.*—Competitions in this column are exclusively for children. Answers must be accompanied by the age of competitor.

*Three marks each*—Gem, Daisy. *Two marks*—Fern.

### Letters.

This week I publish letters on St. Paul's Cathedral and Carisbrooke Castle with which I am very much pleased.

From "Skye."

St. Paul's Cathedral is said to have been restored by Ethelbert and Sebert, Kings of the East Angles, on the site of a Christian church which existed in the time of the Romans. When Sir Christopher Wren, the architect of the present Cathedral, was laying the foundations, he found relics of three different ages at three successive depths beneath the site of the church: firstly, Saxon coffins and tombs; secondly, British graves, with the wooden and ivory pins which fastened the shrouds of those who lay in them; and thirdly, Roman lamps, lacrymatories, and urns, proving the existence of a Roman cemetery on the spot. Old St. Paul's has been burnt five times, the last time being in the year 1666, in the reign of Charles II., when the Great Fire broke out over London and burnt it nearly all down. A very interesting book written by Ainsworth, called "Old St. Paul's," tells all about the Plague and the Great Fire. Old St. Paul's had attained its greatest magnificence in the thirteenth century, when it was a vista of Gothic arches 700 feet in length. At the east end was the shrine of St. Erkenwald, its fourth bishop, son of King Offa, containing a great sapphire which had the reputation of curing diseases of the eye. It was in old St. Paul's that King John acknowledged the supremacy of the Pope; and here the first English martyr, William Sawtre, was stripped of his priestly vestments before being sent to the stake at Smithfield. Early in the sixteenth century it was desecrated to such an extent as to have become more a house of merchandise, usury, horse fairs, brawls, etc., than a church, and the middle aisle of the nave, called "Paul's Walk," was the fashionable promenade of London, where all the dandies met and used it as a club-room to talk and lounge in, and "Paul's walkers" was the popular name for the "young men about town." The great bell of St. Paul's, which hangs in the south tower, bears the inscription "Richard Phelps made me 1716." It tolls only on the deaths or funerals of the Royal Family, Bishops of London, Deans of St. Paul's, and Lord Mayors.

"Gem's" Letter on St. Paul's.

St. Paul's Cathedral was restored by Ethelbert A.D. 604; it was burnt to the ground in 1087, and in the same year Bishop Maurice founded a second one. King John of France made an offering in the Cathedral at the shrine of St. Erkenwald. A mob in 1336 dragged Walter de Stapleton, Bishop of Exeter, from the altar in the Cathedral to be murdered in Cheapside. Jane Shore did penance there 1484. Wicliffe was also tried for his doctrines in it. A choir of singers on great festivals in Queen Mary's reign used to sing anthems, after vespers, high up aloft in the spire. As early as the 14th century, its cloisters, etc., were used for a thoroughfare; such kinds of business as lotteries, money changing, lawyers' meetings, ballad singing, horse dealing, usury, advertisements stuck on the doors of the Cathedral, and lounging of all sorts being freely carried on there; and things became worse and worse, till its burning in 1660. In 1600 Bankes and his famous horse mounted to the top of it. In Charles's civil wars the Parliamentarians made it a magazine for arms. In the Great Plague in 1665 it was used as a pest house, with about three hundred pallets on its floors. In September, 1666, it was totally burnt, and many people think that this was a judgment and a purging from God because of all the secular purposes it was used for. The present Cathedral was begun by Sir Christopher Wren 1675, and finished 1710. The only remaining part of old St. Paul's is a crypt running the whole way underneath the Cathedral. The dome, with its supporting piers, covers upwards of half an acre, the top of the dome is crowned successively by a lantern, a ball, and a cross, and its height is 365 feet. The west front is approached by a double flight of stairs of Black Man marble. There are beautiful carvings of fruit and flowers in the choir carved by Grinling Gibbons. In the vaults were buried, among others, Sir Christopher Wren, Sir Joshua Reynolds, Lord Nelson, Lord Collingwood, Sir Thomas Laurence, J. M. W. Turner, and the Duke of Wellington. Lord Nelson's remains are in a coffin made out of the main mast of *L'Orient*, and enclosed in a marble sarcophagus made for Cardinal Wolsey. In 1872 a thanksgiving service for the recovery of the Prince of Wales was held in the Cathedral. Every first Thursday in June all the charity children meet there for a service, and this is one of the most wonderful sights in the world. Haydn, the composer, said that he never felt the influence of music so deeply on him as when all those voices sang together.

Daisy of "The Eda" writes on Carisbrook Castle:

Carisbrook Castle is a very interesting and picturesque ruin, although not so perfect as some other of our English ruins. In ancient times Carisbrook was most likely the capital of the Island, and the castle the residence of the lords and captains who administered its government. Some say its name is derived from Whitgara-burgh, the town or city of Whitgara, or Whitga, a Saxon chief; but more likely it came from "Cær," the British for stronghold, and "brook," from the stream which flows through the valley. Some say from this meaning that it is far older than Saxon times, and indeed, from the hill on which the Castle stands, the British might very likely have chosen it for a fortress. However, the present castle does not date later than the Normans, and is built in the style of their military architecture, although it is very likely there might have stood a Saxon tower on the same site. That part of the chamber in which King Charles I. the martyr was confined is shown, and the window from which the poor king tried so hard to escape. The Chapel of St. Nicholas is quite modern. It was rebuilt by George II. on the site of the old edifice in 1738. In the castle yard there is a well said to be 300 feet deep. This is supposed to have been constructed by the Romans, who had possession of the Island in the reign of the Emperor Claudius. Water is obtained by means of a large wheel which is worked by an ass. This is one of the sights of the castle. It is a good mile's walk round the outer wall, the views from which are beautiful indeed. But the chief interest of Carisbrook Castle to us is that Charles I. was prisoner there for some time before he was beheaded. He tried twice to escape from his prison. The first attempt might have been successful, and it is said that Cromwell himself would not have been displeased had the king escaped then, but there was no ship, and no proper means supplied. The second time he nearly did escape; and Cromwell wrote to Hammond that the "King had attempted to get out of his window; and that he had a cord of silk whereby to slip down, but his breast was so big that the bar could not give him passage." After this attempt the poor king's walks "upon the beautiful green ramparts looking out on the sea beyond the fertile valleys of Carisbrook" were stopped, and the royal prisoner had no other view than from his own barred windows, so it is said, looking into the courtyard beneath. Charles I. was not long at Carisbrook after this, as he was removed to Hurst Castle, a dreary fortress on the coast of Hampshire. Poor King Charles! A pretty little story is related of him, that one day in November, as he was going to his prison in Carisbrook a lady presented him with a damask rose which had bloomed in her garden, late in the season as it was. The King was very gratified, and indeed he often received many little acts of kindness from the common sort of people, which cheered him on his sad, dreary way to the scaffold.

**Notice to all Correspondents.**—N.B.—All letters referring to this page, which do not arrive at Norfolk House, Norfolk Street, Strand, W.C. by the *first post on Thursdays*, and are not addressed PRIZE EDITOR, will in future be disqualified and disregarded.

No one will be permitted to win the Monthly or Quarterly Prizes twice in any one year, the annual prizes being offered especially to prevent this.

